# Global diversity patterns in sandy beach macrofauna: a biogeographic analysis

**DOI:** 10.1038/srep14515

**Published:** 2015-09-28

**Authors:** Francisco Rafael Barboza, Omar Defeo

**Affiliations:** 1 Unidad de Ciencias del Mar (UNDECIMAR), Facultad de Ciencias, Iguá 4225, 11400 Montevideo, Uruguay and Grupo de Estudios Pesqueros y de Impacto Ambiental (GEPEIA), Centro Universitario de la Región Este, Ruta nacional N° 9 intersección con Ruta N° 15, Rocha, Uruguay

## Abstract

Unlike the advances generated on land, the knowledge of global diversity patterns in marine ecosystems is limited to a small number of studies. For sandy beaches, which dominate the world’s ocean shores, previous meta-analyses highlighted the role of beach morphodynamics in explaining species richness patterns. Oceanographic variables and historical processes have not been considered, even though they could be main predictors of community structure. Our work, based on 256 sandy beaches around the world, analysed species richness considering for the first time temperature, salinity and primary productivity. Biogeographic units (realms, provinces and ecoregions) were used to incorporate historical factors in modelling processes. Ecoregions, which implicitly include isolation and coastal complexity among other historical geographic factors, best represented trends in species richness worldwide. Temperature was a main predictor of species richness, which increased from temperate to tropical sandy beaches. Species richness increased with tide range and towards wide beaches with gentle slopes and fine grains, which is consistent with the hypothesis that habitat availability has an important role in structuring sandy beach communities. The role of temperature and habitat availability suggests that ocean warming and sea level rise could affect the distribution of obligate species living in these narrow ecosystems.

The understanding of the distribution of life on earth is a main goal in ecology and biogeography^1^,^2^. The accelerated loss of habitat, the increased exploitation of natural populations and the global effects of climate change on life, require greater efforts to analyze diversity patterns worldwide^3^,^4^. Diversity patterns on land have been extensively studied, and a large theoretical framework has been developed on the basis of a clear set of hypotheses supported by empirical evidence^1^,^5^. The increase in species richness towards the tropics has been corroborated for a large number of taxa, identifying temperature, primary productivity, habitat availability and seasonal climate variability as major predictors of observed patterns at a global scale^1^. In contrast, our knowledge of global marine diversity patterns is limited to a small number of studies that have analysed species richness patterns and its underlying predictors, relying on hypotheses mainly generated for terrestrial ecosystems^6^,^7^,^8^.

Much of the information and hypotheses about global patterns of species richness on sandy beaches has been generated over the past two decades^9^,^10^,^11^,^12^. One of the main paradigms derived from meta-analyses is the increase of species richness from microtidal reflective beaches, which are narrow and have steep slopes and coarse sands, to macrotidal dissipative systems characterized by a wide intertidal, fine sands and flat beach slopes^13^. It is argued that harsh reflective environments, where turbulent hydrodynamic regimes prevail, allow the settlement and development of populations belonging to only a few taxa (mostly crustaceans), particularly at supralittoral beach levels^13^. Thus, sandy beach communities are thought to be mainly structured by their physical environment. Additionally, using beach slope (a conservative proxy of the beach morphodynamic state) as covariate, recent studies showed higher species richness in tropical zones than in sub-tropical, warm temperate and cold temperate ones^12^,^13^. Surprisingly, the role of sea surface temperature, sea surface salinity (from now on simply referred to as temperature and salinity) and primary productivity has not been quantitatively considered in the explanation of global diversity patterns in these ecosystems, even though they have been indicated as main predictors of biogeographic structure and species richness for coastal and shelf benthic marine groups^7^,^14^.

Geological and evolutionary histories have been also identified as important drivers of coastal diversity patterns worldwide^7^. Thus, the inclusion of oceanic basins^7^ or other biogeographic units in ecological models has been suggested in order to consider historical processes in the understanding of species richness patterns. In addition, biogeographic information is increasingly considered in conservation planning, since it gives a natural spatial framework within which to implement management actions^15^,^16^. A major step in elucidating global patterns on sandy beaches and generating conservation plans for one of the most threatened ecosystems in the world^17^,^18^, should include a rigorous biogeographic classification in the modelling process.

In this paper we analysed global patterns in species richness on sandy beaches, considering, for the first time, temperature, primary productivity and salinity (in addition to tide range and morphodynamic variables) as explanatory variables of potential trends. We contrasted classical macroecological hypotheses and deepened the understanding of observed latitudinal patterns, until now only addressed by including latitude as an aggregate variable. We evaluated four hypotheses that could be used to explain worldwide diversity patterns on sandy beaches: 1) The Kinetic Energy or Temperature Hypothesis^19^,^20^,^21^, which predicts a positive correlation between species richness and temperature on the assumptions that: a) higher speciation rates are expected in warmer conditions as a consequence of increased metabolic rates; or b) thermal tolerance defines species distribution ranges, particularly for ectotherms, with more species tolerant of tropical conditions. 2) The Potential Energy Hypothesis^19^,^22^, which states that more productive areas promote the coexistence of a larger number of species. 3) The Swash Exclusion Hypothesis^23^, which states that the swash climate experienced by the macrofauna on the beach face is closely coupled to beach type, predicting a decrease in species richness towards beaches characterized by coarse sands, steep slopes and a harsh swash (i.e. water movement over the beach face, after a broken wave collapses on the sand). 4) The Habitat Availability Hypothesis, as formulated for sandy shores^13^,^23^, which states that increasing tide range (defined as the vertical difference between the high tide and the succeeding low tide, and used here as a proxy for habitat availability) positively influences species richness. To this end, we analysed the macrofaunal species richness information from more than 250 sandy beaches around the world (Fig. 1), using Generalized Additive Mixed Models (GAMM) and Generalized Linear Mixed Models (GLMM). We included domains, provinces and ecoregions defined in the Marine Ecoregions of the World (MEOW) nested system^16^ as random intercepts, in an effort to account for potentially different signatures of evolutionary history among biogeographic units.

## Results

### General trends

Ecoregions were the most informative units in explaining worldwide trends in sandy beach species richness (Table 1). Tropical ecoregions Guayaquil, Panama Bight, Gulf of Oman and Western Arabian Sea showed the highest species richness, with mean values ranging from 21 to 33 (Fig. 2). The temperate ecoregions defined by Central New Zealand, North Sea, Northeastern New Zealand, Oregon Washington, Vancouver Coast Shelf, Rio Grande, South European Atlantic Shelf and the tropical ecoregions Central and Southern Great Barrier Reef, and Southeast Madagascar have also had a high number of sandy beach macrofaunal species (Fig. 2).

Species richness decreased with grain size (Fig. 3a) and beach slope (Fig. 3b) and increased with tide range (Fig. 3c). These patterns, taken together, mean that the richest beaches were dissipative and exhibited larger tide ranges. Species richness also increased with temperature (Fig. 3d), highlighting the greatest richness exhibited by tropical sandy beaches. Salinity and primary production were not significant explanatory variables.

### Global patterns

In GAMM, the most parsimonious random intercept structure included only ecoregions. This random structure’s Akaike’s Information Criterion (AIC) was almost 2 points lower than the one that included a random intercept with a nested structure between provinces and ecoregions and in more than 4 points lower in comparison with other random structures or the model without a random structure (Table 1).

Using the ecoregions as random intercepts, we found the optimal fixed component of the model. The final GAMM reached an AIC of 1459.9, being 3.4 points lower than the previous one, thus indicating a better empirical support (sensu Burnham & Anderson^24^) when compared with other models. As a result of the modelling process, the GAMM retained 4 of the 6 variables initially included (Table 2), whose relative importance was, in decreasing order: grain size, beach slope, tide range and temperature. On the link function scale, species richness exhibited linear trends with grain size and temperature (Supplementary Fig. S1a,d online) and non-linear shapes with beach slope and tide range (Supplementary Fig. S1b,c online). Therefore, grain size and temperature were included as linear predictors in the GLMM, whereas beach slope and tide range were included as logarithmic terms (Table 2).

The GLMM obtained (Table 2) exhibited a lower AIC than the most parsimonious GAMM. The results of this model, expressed in the original response variable scale, showed a decrease of species richness with grain size and slope (Fig. 3a,b) and increased with tide range, reaching an upper ceiling at values larger than 3 m (Fig. 3c). Species richness increased with temperature (Fig. 3d). VIF values ranged between 1.04 and 1.77, confirming a lack of any multicollinearity among variables. The obtained GLMM presented a marginal R^2^ (i.e. variance explained only by fixed effects) of 0.61 and a conditional R^2^ (i.e. variance explained by fixed and random effects) of 0.73. The parameters of the GLMM, the associated standard errors and the corresponding statistical significance are presented in Supplementary Table S1.

## Discussion

We analysed the relative contribution of a range of variables in explaining current-day species richness on sandy beaches at a global scale. Our analyses demonstrate the central role of beach morphodynamics and temperature (used for the first time as a global predictor of species richness on sandy beaches) in structuring benthic marine communities in these ecosystems. We also explicitly assessed the role of salinity and primary productivity (see Rodil *et al.*^25^ for a regional approach), with known effects on marine invertebrates^14^,^26^.

Species richness significantly increased in response to decreasing sand particle size and in flatter and wider beaches. Indeed, grain size and beach slope were the main predictors of species richness at a global scale, which increased from reflective to dissipative beaches. This pattern is consistent with the Swash Exclusion Hypothesis, explicitly stated as one of our main working hypotheses. The consistency of these patterns worldwide, and the close relationship between morphodynamics and species richness at regional scales^27^,^28^,^29^,^30^, suggest that species richness on sandy beaches is therefore predictable on the basis of the physical nature of beach environments. These findings are also in agreement with the Habitat Harshness Hypothesis^31^,^32^, formulated at the population level, which states that steeper slopes and coarse sand of reflective beaches promote turbulent hydrodynamic regimes in swash zones and abrasive effects on intertidal species, thereby reducing their feeding times, increasing their investment in maintenance and determining lower fecundity and higher mortality rates in comparison to dissipative beach populations^23^. Therefore, environmental conditions that prevail in reflective beaches represent a strict physical filter that allows the settlement and persistence of certain species, conditioning meta-population and meta-community dynamics among sandy beaches^33^. Only species with life history traits that allow them to resist breaking waves and coarse sand (e.g. crustaceans) are found in reflective sandy beaches^30^. We speculate that the morphodynamic gradient from reflective to dissipative beaches promotes nested patterns at regional scales, i.e. the species composition of less rich communities at reflective beaches is a non-random subset of the species observed in richer dissipative ones^34^, determining biodiversity trends observed worldwide.

Tide range had an important role as predictor of species richness, which is consistent with our main prediction related to the Habitat Availability Hypothesis. The relevance of tide range has not always been considered in sandy beach ecology because most studies had considered sites with similar tide ranges at smaller spatial scales. In our model, tide range was still significant after adjusting for morphodynamic effects (see Table 2), and thus it could be considered as an area variable with utmost importance in explaining macroscale variations in sandy beach diversity patterns. The asymptotic relationship observed between species richness and tide range reminds us the species-area relationship extensively reported on terrestrial ecosystems. Larger tide ranges widen beaches and modify their intertidal profile^35^, increasing habitat heterogeneity and availability, which could allow species coexistence and the settlement of a greater number of species^10^,^13^. Moreover, beaches with larger areas could support larger populations, which could reduce the probability of species extirpation. Our results are consistent with the hypothesis that habitat availability influences species richness in coastal areas^7^.

Data on temperature, primary productivity and salinity allowed us to objectively assess the role of these variables in explaining global patterns in sandy beach macrofauna. Our findings revealed the role of temperature (and a set of correlated variables and processes not considered here, as historical disturbance regimes and seasonality), in structuring sandy beach macrofaunal communities. We showed an increase of species richness from temperate to tropical sandy beaches, which is in agreement with the four tropical ecoregions identified as diversity hot-spots in this work (Guayaquil, Panama Bight, Gulf of Oman and Western Arabian Sea). Other areas poorly represented in our work could also arise as potential hot-spots (e.g., the Western Pacific^7^), but this requires further information. The observed monotonic increase of macrofaunal species richness with temperature is consistent with the Kinetic Energy or Temperature Hypothesis (detailed in the Introduction) and is in agreement with patterns obtained by Tittensor *et al.*^7^ for coastal fishes, non-oceanic sharks, non-squid cephalopods and corals (see also Willig *et al.*^5^ for a general review in other environments). This suggests that higher metabolic rates or relaxed thermal constraints promote diversity^7^, mainly in ectotherms that are most sensitive to temperature variations and constitute the vast majority of obligate sandy beach species.

Salinity and primary productivity were not identified as key explanatory variables of species richness. This is in disagreement with Belanger *et al.*^14^, who identified these variables as main environmental predictors of benthic marine biogeographic structure in coastal and shelf waters. In the case of salinity, only oceanic beaches were included in our database, and therefore the reduced range of variation of this variable could explain its absence in the final models. Alternatively, the coarse scale of salinity measures might obscure local effects of salinity at the level of individual beaches/surf zones. Therefore, this pattern could change drastically if sandy beaches from transitional estuarine systems are included in the global database, as already observed at the scale of a single ecoregion along an entire estuarine gradient^36^. Thus, further hypothesis testing and scientific collaboration through research networks are required to strengthen our database. Concerning primary productivity, our findings are in agreement with previous papers at a global scale that highlighted the minor relevance of this variable in explaining species richness patterns in several ectothermic coastal groups^7^. Hence, our results are not in agreement with the Potential Energy Hypothesis.

Global variations in species richness were explained at the ecoregional scale. The inclusion of the random effect in our model improved the model fit (the random effect improved the variance explained by the model by 12%, see also AIC values in Table 1). What is perhaps more significant, is that this type of information has been seldom considered in sandy beach ecology^37^. It must be highlighted that the reduced dispersal of several sandy beach species, and the consequent increase of isolation among regions, may result in historical factors having a greater influence on diversity patterns than other faunal groups with greater motility ranges (e.g. pelagic species)^7^,^8^. We must highlight that the inclusion of Ecoregions in the models could account for differences in coastal complexities or exposure^16^ that are not explicitly measured in the articles but may have an effect on sandy beach species richness. Our results also provide crucial information for scientists, managers and stakeholders to define areas with precise boundaries (i.e. ecoregions) onto which implement sandy beach conservation strategies^37^.

The retention of temperature in our global model allows sandy beach ecology to hypothesize about potential consequences of global warming and related effects, including sea level rise. Sandy beach communities are physically-controlled^23^ and mainly dominated by marine ectotherms with a low thermal tolerance^38^, making them particularly susceptible to climate change^39^. In this context, the most rapid warming and the concurrent increase in sea level observed in coastal marine ecosystems over the past decades^40^,^41^ could be particularly harmful in sandy beaches, characterized by a linear and very narrow nature of the habitat that defines an absence of spatial refuges or compensatory habitats for obligate beach specialists with a very limited dispersal capacity^39^. Thus, the increase in temperature could turn tropical beaches (the richest on earth as demonstrated here) into extremely warm areas, promoting a poleward range shift of several coastal species^42^ and favouring diversity peaks in temperate regions^43^. At the same time, the extremely high biomass of filter feeders observed in temperate beaches could be eventually threatened by the decrease in their main food source given by temperate surf diatoms, which could be affected by the increasing frequency of El Niño Southern Oscillation^44^. The “tropicalization” (sensu Cheung *et al.*^45^) of sandy beach communities, i.e. an increasing prevalence of species with a tropical biogeographic origin, has been recently supported by long-term and large scale studies in South American beaches that showed mass mortalities of sandy beach filter feeder clams of Antarctic origin as a response of long-term increase in temperature^46^,^47^,^48^,^49^, favouring a demographic explosion of species with warm water preferences^50^,^51^.

This work, based on a comprehensive dataset, demonstrated for the first time the role of temperature (in addition to beach slope, grain size and tide range) in determining global diversity patterns on sandy beaches. Our analysis at a global scale gave new insights about the ecological processes underlying global trends. We also identified ecoregions as the MEOW biogeographic units where processes underlying world diversity changes are probably framed, with important implications for biodiversity conservation. Further questions and gaps in sandy beach ecology remain unexplored. Future work that deconstruct global trends in relation to taxonomy, development mode, feeding mode and other traits are required in order to disentangle diversity drivers on sandy beaches at larger scales. Taking into account the availability of low resolution satellite environmental data for coastal ecosystems, which have been estimated on a different spatial scale from other predictors (measured at the within-beach scale), we encourage the measurement in field (see Smit *et al.*^52^) and the explicit inclusion of temperature, salinity and primary productivity in future sandy beach research, in an effort to enable future meta-analyses.

## Methods

### Database

**Figure 1:**
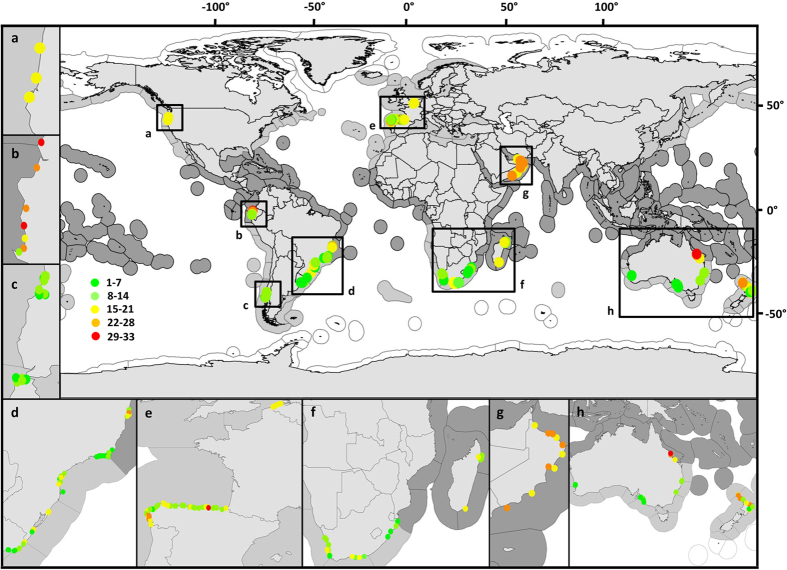
Location of sandy beaches for which data was included in this paper (n = 256). See insets (**a**–**h**) for better resolution. The colour scale indicates the species richness for each beach. Outlined areas around continents indicate the ecoregions defined by Spalding *et al.*^16^, downloaded from http://maps.tnc.org/gis_data.html: white indicates polar ecoregions, light gray temperate ecoregions and dark grey tropical ecoregions. Maps were generated using gvSIG 1.12 (http://www.gvsig.org).

**Figure 2:**
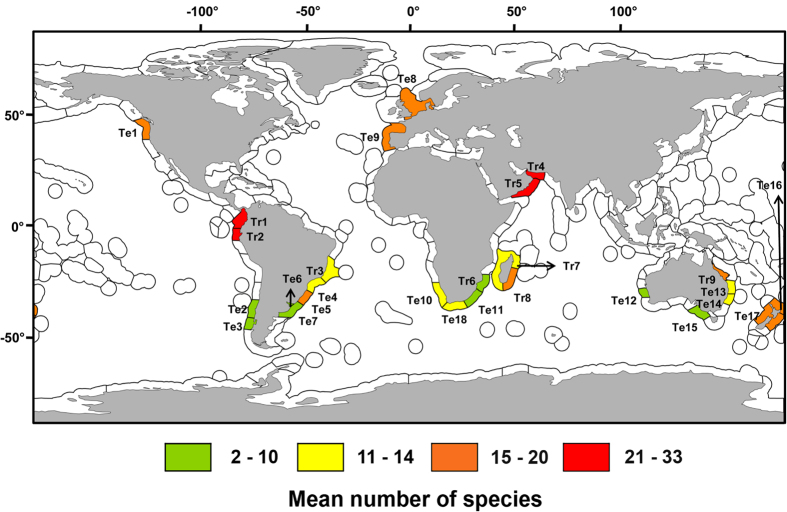
Sandy beach species richness in temperate (Te) and tropical (Tr) ecoregions defined in the MEOW system developed by Spalding *et al.* ^**16**^. Te1: Oregon, Washington, Vancouver Coast and Shelf; Te2: Araucanian, Te3. Chiloense; Te4: Southeastern Brazil; Te5: Rio Grande; Te6: Rio de la Plata; Te7: Uruguay-Buenos Aires Shelf; Te8: North Sea; Te9: South European Atlantic Shelf; Te10: Namaqua; Te11: Natal; Te12: Houtman; Te13: Tweed-Moreton; Te14: Manning-Hawkesbury; Te15: Western Bassian; Te16: Northeastern New Zealand; Te17: Central New Zealand; Te18: Agulhas Bank; Tr1: Panama Bight; Tr2: Guayaquil; Tr3: Eastern Brazil; Tr4: Gulf of Oman; Tr5: Western Arabian Sea; Tr6: Delagoa; Tr7: Western and Northern Madagascar; Tr8: Southeast Madagascar; Tr9: Central and Southern Great Barrier Reef. The map containing ecoregions was downloaded from http://maps.tnc.org/gis_data.html. The final map was generated using gvSIG 1.12 (http://www.gvsig.org).

**Figure 3:**
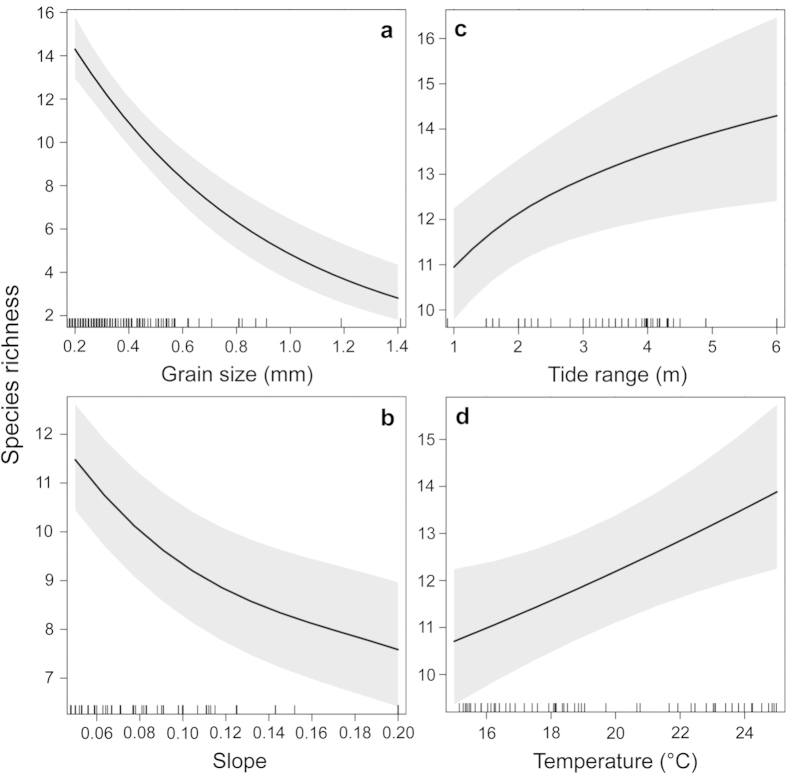
Generalized Linear Mixed Model (GLMM, expressed on the original response variable scale) relating species richness and environmental predictors for the 256 sandy beaches on five continents. Mean partial effects of grain size (**a**), beach slope (**b**), tide range (**c**) and temperature (**d**) on species richness are shown (solid line). Gray shadows indicate 2 times the standard error. The marks on the x-axis show the distribution of measured values for each predictor.

Our database included information on benthic macrofaunal species richness from more than 250 sandy beaches around the world (Fig. 1). These data were based on the information contained in Defeo & McLachlan^13^ and on new information gathered from the literature. Using Scopus, Jstor and Google Scholar search engines, we reviewed articles and theses about sandy beaches, collecting information of species richness (i.e. the total number of species surveyed) and environmental variables (see details below) only when this information was explicitly included in the main text or in the supplementary material. We did not approximate estimates from figures.

Manuscripts were assessed and, when raw data were available, results were verified. We took into account the following cautionary criteria: (1) following Schoeman *et al.*^53^, we tried to ameliorate the effects of sampling effort by considering only those studies that sampled the entire beach width (see Supplementary Table S2 online); in those studies where subtidal samples were taken, these data were not used; (2) when monthly or seasonal surveys were present in the data sources, the species pool collected across all samples reported was used to provide only one estimate for each beach; (3) only marine species were considered, excluding terrestrial forms. This was because most articles available in the literature do not consider terrestrial species like insects or arachnids, surveys on sandy beaches rarely take into account the landward distribution of this species and most sampling devices used (e.g. quadrats) underestimate the number of insect species, since they are ineffective at capturing highly mobile organisms^54^. The final screening reduced our dataset to 256 sandy beaches from five continents.

Beach slope, grain size and tide range were selected a priori as the key sandy beach physical variables gathered from the literature, based on relevant findings earlier provided by Defeo and McLachlan^13^ (and references therein). Moreover, these were the only variables available in all the articles considered in this work. Temperature, salinity and primary productivity were also explicitly included in the database. Information about these variables was gathered from databases available online. After geographically locating the beaches, mean annual temperature and salinity were obtained from the World Ocean Atlas 2009 1° grid cell objectively averaged dataset^55^,^56^, which is considered the most reliable for the purposes and scale of our study^14^. Mean annual primary productivity, with a 0.5° grid cell resolution, was obtained from AquaMaps environmental dataset^57^, since it was not available in the World Ocean Atlas 2009 database. Sampling effort was gathered from the revised articles, and the relationship between this variable and species richness was assessed. Some beaches were excluded from the plot because: 1) effort data was lacking; or 2) exhibited extreme values of species richness or sampling effort that made plot visualization difficult and, at the same time, did not modify the general trend. The relationship between species richness and sampling effort for a subset of beaches (n = 240, Supplementary Fig. S2 online) was not significant (r^2^ = 0.0029; p = 0.40) and allowed us to discard this variable as a predictor in the modelling process. This allowed us to discard the inclusion of “study” (which is very related to sampling effort) as a random effect in the model.

Using the maps (http://conserveonline.org) provided by Spalding *et al.*^16^ for the MEOW system for shelf and coastal areas, we assigned each beach to an ecoregion, a province and a realm. We found beaches for 9 realms, 18 provinces and 27 ecoregions that fulfilled our search criteria.

### Data analysis

GAMM were implemented using the gamm4 R package, to determine the effect and the relative importance of environmental variables (beach slope, grain size, tide range, temperature, salinity and primary productivity) on species richness. The Poisson distribution and a log-link function were used for fitting. Differences in species richness among beaches could not be attributed only to the environmental variables assessed, but also to past geomorphological changes and the consequent evolutionary histories in isolation that may have promoted the biogeographical subdivision recognized. Therefore, we adjusted this source of variation by including the units defined by Spalding *et al.*^16^ as random intercepts. Mixed models allowed us to include factor variables with several levels (9 realms, 18 provinces, 27 ecoregions and their nested structures), without losing a great amount of degrees of freedom, and in consequence statistical power, in the assessment of environmental predictors. After including all potential predictors in the fixed component of the model, we selected the most parsimonious random intercept using the Akaike’s Information Criterion (AIC), considering nested and non nested structures (see details in Results). Once the optimal random structure was obtained, we found the optimal fixed structure of the model. Environmental predictors were included as smooth terms using penalized regression splines with up to 3 degrees of freedom^58^. Submodels were obtained by eliminating variables, until the model that included all significant variables with the lowest AIC was accomplished. In order to obtain a parametric version, model coefficients were estimated by a GLMM, using lme4 R package, substituting non-parametric functions by similar parametric ones. Collinearity was checked using the variance inflation factor (VIF) of the package car. VIF values >4 were considered as evidence of collinearity, i.e. the information carried by a predictor having such a VIF is contained in a subset of the remaining predictors. The marginal R^2^ and the conditional R^2^ of the obtained GLMM were calculated using the MuMIn package^59^. In the final GAMM and GLMM, residuals were checked.

## Additional Information

**How to cite this article**: Barboza, F.R. and Defeo, O. Global diversity patterns in sandy beach macrofauna: a biogeographic analysis. *Sci. Rep.* **5**, 14515; doi: 10.1038/srep14515 (2015).

## Supplementary Material

Supplementary Information

## Figures and Tables

**Table 1:** Random intercept selection based on Akaike’s Information Criterion (AIC) for global models.

Random intercept	AIC
Without random intercepts	1510.8
Realms	1486.4
Provinces	1477.6
Ecoregions	1467.3
Realms|Provinces	1478.4
Provinces|Ecoregions	1469.2
Realms|Provinces|Ecoregions	1471.2

Note that we also include the AIC value of the model without random intercept. Those random intercepts that include more than one biogeographic unit suppose nested structures, where the leftmost unit nests the others.

**Table 2:** Fixed component selection for Generalized Additive Mixed Models (GAMM) relating species richness and environmental predictors for 256 sandy beaches around the world.

Model type	Model expression	AIC
GAMM	s(Grain size, 1) + s(Slope, 1.72) + s(Temperature,1) + s(Tide Range, 2.53) + s(Productivity, 1) + s(Salinity,1)	1467.3
	s(Grain size, 1) + s(Slope, 1.72) + + s(Temperature,1) + s(Tide Range, 2.53) + s(Productivity, 1)	1463.3
	s(Grain size, 1) + s(Slope, 1.69) + + s(Tide Range, 2.42) + s(Temperature,1)	1459.9
GLMM	Grain size + log(Slope) + log(Tide range) + Temperature	1445.2

Main models obtained from the modelling process are expressed in R language. Numbers presented in the model expression refer to the degrees of freedom estimated for smooth terms (s). Akaike’s Information Criterion (AIC) values are given. All the terms of the last GAMM were significant (p < 0.05). The parametric version of the most parsimonious GAMM is included (GLMM). Estimated coefficients of the GLMM are presented in Table S1.
