# Supplementary material for: Global diversity patterns in sandy beach macrofauna: a biogeographic analysis
Source: Sci Rep. 2015 Sep 28;5:14515. doi: 10.1038/srep14515 (PMC4585946; doi:10.1038/srep14515)
Supplement: Supplementary Information [file srep14515-s1.pdf]

Supplementary information

Global diversity patterns in sandy beach macrofauna: a biogeographic analysis

Francisco Rafael Barboza & Omar Defeo*

Unidad de Ciencias del Mar (UNDECIMAR), Facultad de Ciencias, Iguá 4225, 11400 Montevideo, Uruguay and Grupo de Estudios Pesqueros y de Impacto Ambiental (GEPEIA), Centro Universitario de la Región Este, Ruta nacional Nº 9 intersección con Ruta Nº 15, Rocha, Uruguay

*Correspondence and requests for materials should be addressed to O. D. (odefeo@dinara.gub.uy)

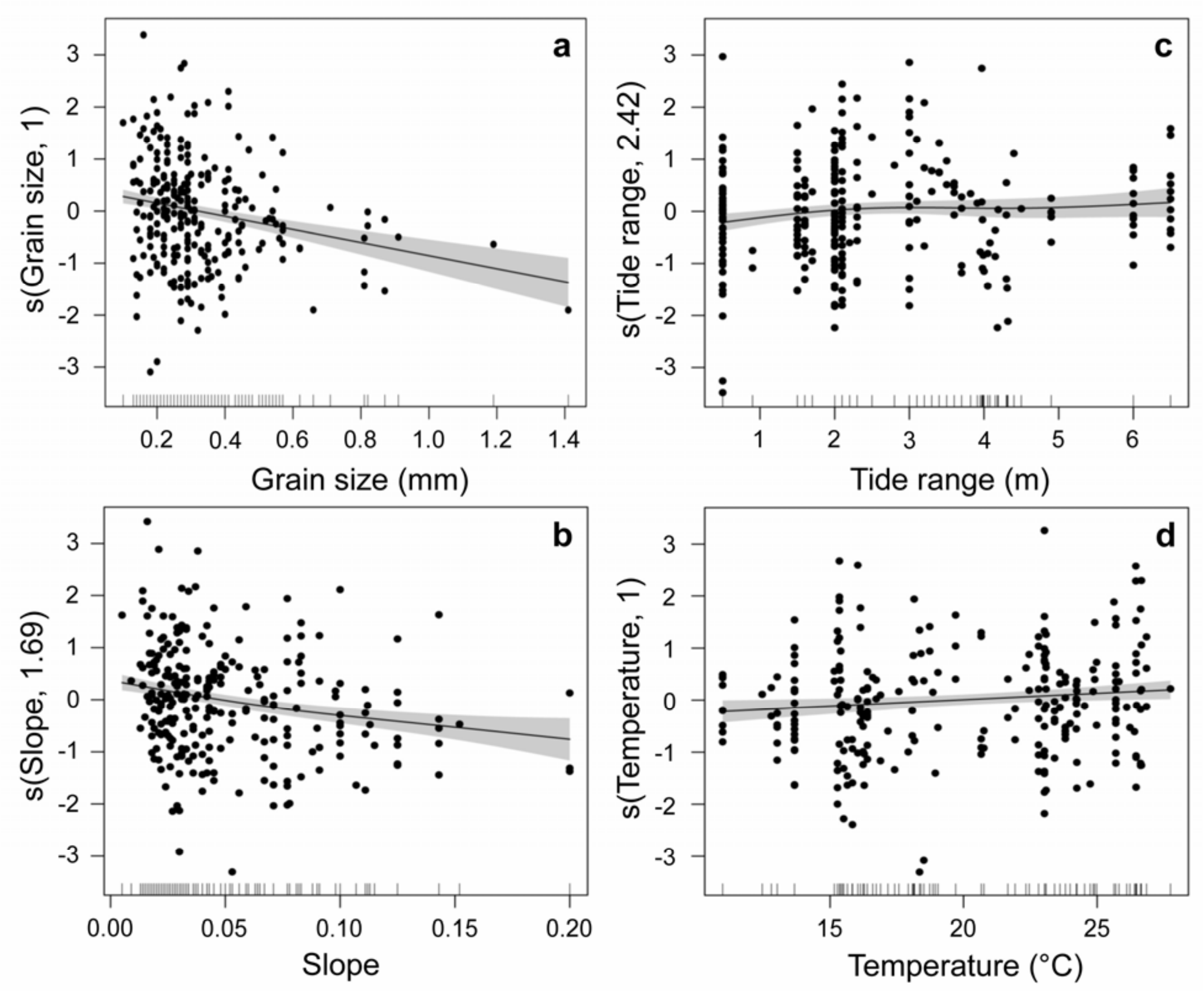

Figure S1. Best Generalized Additive Mixed Model (GAMM, expressed on the link function scale) relating species richness and environmental predictors for the 256 sandy beaches on five continents. Mean partial effects of grain size (a), beach slope (b), tide range (c) and temperature (d) on species richness are shown (solid line). Gray shadows indicate 2 times the standard error. The marks on the x-axis show the distribution of measured values for each predictor.

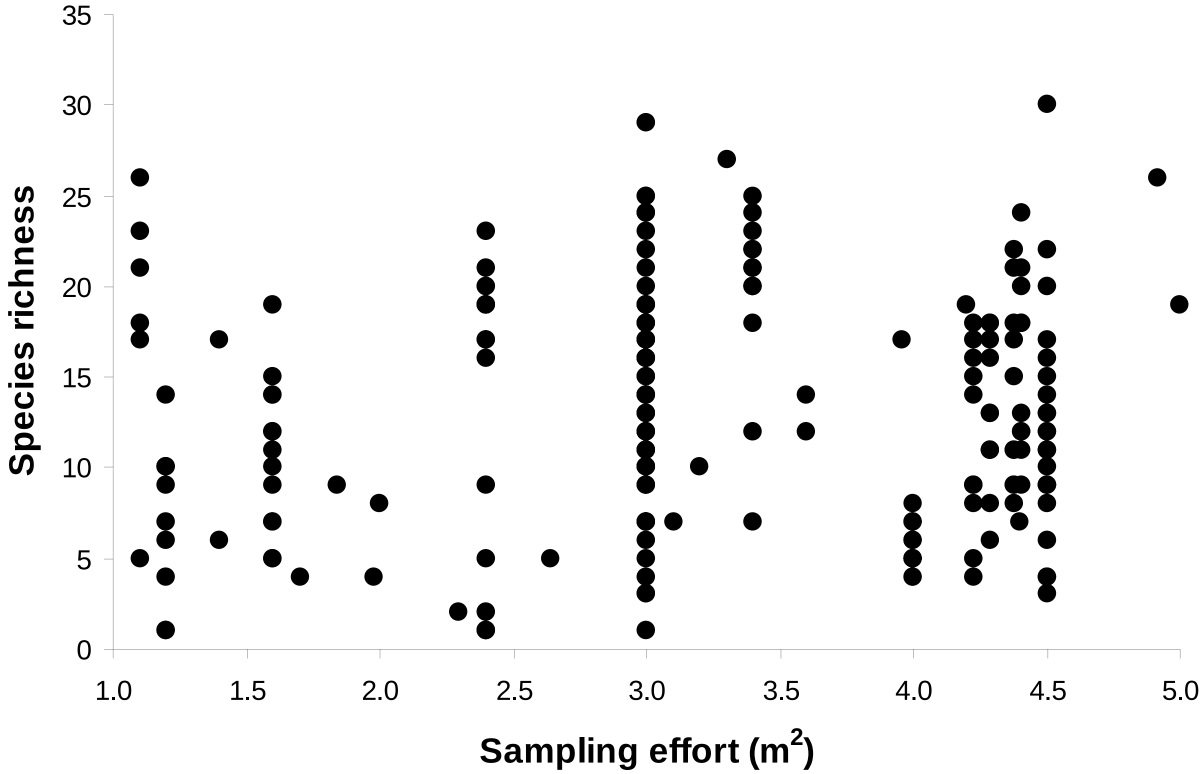

Figure S2. Species richness against sampling effort for a subset of 240 sandy beaches around the world. The linear equation was Y = 12.00 + 0.34X (r^2 = 0.0029, p = 0.40).

Table S1. Fixed effects of the Generalized Linear Mixed Model (GLMM) relating species richness and environmental predictors for the 256 sandy beaches included in the database. Note that slope and tide range were included as logarithmic terms. SE: Standard Error. **p < 0.01, ***p < 0.001.

Fixed term	Estimate	SE
Intercept	1.37***	0.28
Temperature	0.03**	0.01
Grain size	-1.36***	0.20
log (Tide range)	0.15**	0.05
log (Slope)	-0.30***	0.05

Table S2. Summary information of sandy beach surveys identified from the literature reviewed. Values of total sampling effort per sampling event were approximated from the reference sources. In cases when 2 or more beaches were sampled, variability in sampling effort among beaches reflects the area of the beach (from the supralittoral to the lower level of the swash zone) available for sampling. The same holds true when more than one sampling event was conducted.

Country	Number of beaches surveyed	Sampling events per beach	Total sampling effort (m^2) per beach and sampling event	Total number of species collected	References
Australia. South Africa and US	9	1	2.4	1-20	1 [a]
Australia	10	1	3.0	4-17	2
Australia	6	1	4.5	9-30	3
Australia	25	1	3.0	3-29	4
Belgium	8	1	2.4	19-23	5
Brazil	2	12	1.1	23-26	6
Brazil	10	1	1.4-1.8	7-19	7 [b]
Brazil	1	14	2.4	17	8
Brazil	1	3	1.1	5	9
Brazil	2	9	1.4	6-17	10, 11
Brazil	1	1	5	19	12, 13
Brazil	1	2	3.6	14	14
Brazil	3	5	1.1	17-21	15, 16
Brazil	1	2	2.0	8	17 [c]
Brazil	1	13	3.3	27	18
Brazil	3	8	1.6	5-7	19
Brazil	15	2	4.0	4-8	20
Brazil, Chile, Madagascar, South Africa	52	1	3.3-4.5	4-24	21
Chile	6	1	4.5	3-11	22
Chile	10	1	1.2	1-14	23
Ecuador	7	1	1.9-4.9	9-33	24
New Zealand	9	1	3.0	1-25	25 [d]
Oman	10	1	3.4	18-25	26
South Africa	3	1	15.8-17.5	12-21	27 [e]
South Africa	2		3.0	10-12	28
Spain	18	1	3.0	10-24	29 [f]
Spain	6	1	N/A	5-27	30
Spain	19	1	3.0	9-29	31
Uruguay	5	1	1.7-4.0	2-17	32
Uruguay	10	11	3.0-4.2	7-19	33

[a] Qualitative collections were made over the shore for about 30 min per beach to record rare species that might have been missed.
[b] The analysis was restricted to the intertidal level, avoiding information of the truly sublitoral fringe.
[c] Only 1 from a total of 2 beaches was considered after assessing the information.
[d] In McLachlan & Dorvlo[34].
[e] Additional environmental information for these beaches were gathered from McArdle & McLachlan[35].

[f] Only 18 from a total of 34 beaches were considered in the analysis.

REFERENCES

1. McLachlan, A. Dissipative beaches and macrofauna communities on exposed intertidal sands. *J. Coast. Res.* **6,** 57–71(1990).

2. Hacking, N. Macrofaunal community structure of beaches in northern New South Wales, Australia. *Mar. Freshw. Res.* **49,** 47–53 (1998).

3. McLachlan, A., De Ruyck, A. M. C. & Hacking, N. Community Structure on sandy beaches: patterns of richness and zonation in relation to tide range and latitude. *Rev. Chil. Hist. Nat.* **69,** 451–467 (1996).

4. Hacking, N. Effects of physical state and latitude on sandy beach macrofauna of eastern and southern Australia. *J. Coast. Res.* **23,** 899–910 (2007).

5. Degraer, S., Volckaert, A. & Vincx, M. Macrobenthic zonation patterns along a morphodynamical continuum of macrotidal, low tide bar/rip and ultra-dissipative sandy beaches. *Estuar. Coast. Shelf Sci.* **56,** 459–468 (2003).

6. Silva, P. S. R, Neves, L. P. & Bemvenuti, C. E. Temporal variation of sandy beach macrofauna at two sites with distinct environmental conditions on Cassino beach, extreme Southern Brazil. *Brazilian J. Oceanogr.* **56,** 257–270 (2008).

7. Borzone, C. A., Souza, J. R. B. & Soares, A. G. Morphodynamic influence on the structure of inter and subtidal macrofaunal communities of subtropical sandy beaches. *Rev. Chil. Hist. Nat.* **69,** 565–577 (1996).

8. Souza, J. R. B. *Produção secundária da macrofauna bentônica da Praia de Atami – PR*, PhD Thesis, Universidade Federal do Paraná (1998).

9. Calliari, L. J., Klein, A. H. F. & Barros, F. C. R. Beach differentiation along the Rio Grande do Sul coastline (southern Brazil). *Rev. Chil. Hist. Nat.* **69,** 485–493 (1996).

10. Alves, E. S. & Pezzuto, P. R. Effect of cold fronts on the benthic macrofauna of exposed sandy beaches with contrasting morphodynamics. *Brazilian J. Oceanogr,* **57,** 73–96 (2009).

11. Alves, E. S. & Pezzuto, P. R. Effect of morphodynamics on annual average zonation pattern of benthic macrofauna of exposed sandy beaches in Santa Catarina, Brazil. *Brazilian J. Oceanogr.* **57,** 189–203 (2009).

12. Gianuca, N. M. in *Sandy beaches as ecosystems* (ed. McLachlan, A. & Erasmus, T.) 413–419 (Dr. W. Junk Publishers, The Hague, 1983).

13. Gianuca, N. M. *The ecology of a sandy beach in Southern Brazil*, PhD Thesis, University of Southampton (1985).

14. Lepka, D. L. *Macrofauna de praias arenosas com diferentes graus de morfodinamismo no parque estadual da Ilha do Cardoso, SP, Brasil,* MSc Thesis, Universidade Federal do Paraná (2008).

15. Neves, F. M. & Bemvenuti, C. E. Spatial distribution of macrobenthic fauna on three sandy beaches from northern Rio Grande do Sul, Southern Brazil. *Brazilian J. Oceanogr.* **54,** 135–145 (2006).

16. Neves, F. M. & Bemvenuti, C. E. Variabilidade diária da zonação da macrofauna bentônica em praias arenosas do litoral norte do Rio Grande do Sul. *Iheringia, Sér. Zool.* **99,** 71–81 (2009).

17. Ramalho Fernandes, R. S. & Soares-Gomes, A. Community structure of macrobenthos in two tropical sandy beaches with different morphodynamic features, Rio de Janeiro, Brazil. *Mar. Ecol.* **27,** 160–169 (2006).

18. Souza, J. R. B. & Gianuca, N. M. Zonation and seasonal variation of the intertidal macrofauna on a sandy beach of Parana state, Brazil. *Sci. Mar.* **59,** 103–111 (1995).

19. Veloso, V. G. & Cardoso, R. S. Effects of morphodynamics on the spatial and temporal variation of macrofauna on three sandy beaches, Rio de Janeiro State, Brazil. *J. Mar. Biol. Assoc. UK* **81,** 369–375 (2001).

20. Veloso, V. G., Caetano, C. H. S. & Cardoso, R. S. Composition, structure and zonation of intertidal macrofauna in relation to physical factors in microtidal sandy beaches in Rio de Janeiro State, Brazil. *Sci. Mar.* **67,** 393–402 (2003).

21. Soares, A. G. *Sandy beach morphodynamics and macrobenthic communities in temperate, subtropical and tropical regions: A macroecological approach*, PhD Thesis, University of Port Elizabeth (2003).

22. Jaramillo, E., Duarte, E. & Contreras, H. Sandy beach macroinfauna from the coast of Ancud, Isla de Chiloé, southern Chile. *Rev. Chil. Hist. Nat.* **73,** 771-786 (2000).

23. McLachlan, A., Jaramillo, E., Donn, T.E. & Wessels, F. Sand beach macrofauna communities and their control by the physical environment: A geographical comparison. *J. Coast. Res.* **15,** 27–38 (1993).

24. Vanagt, T. *The role of swash in the ecology of Ecuadorian sandy beach macrofauna, with special reference to the surfing gastropod Olivella semistriata*, PhD Thesis, Ghent University (2007).

25. Stephenson, G. & McLachlan, A. Community responses of the macroinfauna to physical factors on some wave-exposed sandy beaches in northern New Zealand. Unpublished.

26. McLachlan, A., Fisher, M., Al-Habsi, H. N., Al-Shukairi, S. & Al-Habsi, A. M. Ecology of sandy beaches in Oman. *J. Coast. Conserv.* **4,** 181–190 (1998).

27. Schoeman, D.S., Wheeler, M. & Wait, M. The relative accuracy of standard estimators for macrofaunal abundance and species richness derived from selected intertidal transect designs used to sample exposed sandy beaches. *Estuar. Coast. Shelf Sci.* **58S,** 5–16 (2003).

28. Harris, L., Nel, R., Smale, M. & Schoeman, D. Swashed away? Storm impacts on sandy beach macrofaunal communities. *Estuar. Coast. Shelf Sci.* **94,** 210–221 (2011).

29. Lastra, M *et al.* Ecology of exposed sandy beaches in northern Spain: Environmental factors controlling macrofauna communities. *J Sea Res.* **55,** 128–140 (2006).

30. Rodil, I. F., Lastra, M. & López, J. Macroinfauna community structure and biochemical composition of sedimentary organic matter along a gradient of wave exposure in sandy beaches (NW Spain). *Hydrobiologia* **579,** 301–316 (2007).

31. Rodil, I. F., Lastra, M. & Sánchez-Mata, A. G. Community structure and intertidal zonation of the macroinfauna in intermediate sandy beaches in temperate latitudes: North coast of Spain. *Estuar. Coast. Shelf Sci.* **67,** 267–279 (2006).

32. Defeo, O., Jaramillo, E. & Lyonnet, A. Community structure and intertidal zonation of the macroinfauna in the Atlantic coast of Uruguay. *J. Coast. Res.* **8,** 830–839 (1992).

33. Lercari, D. & Defeo, O. Large-scale diversity and abundance trends in sandy beach macrofauna along full gradients of salinity and morphodynamics. *Estuar. Coast. Shelf Sci.* **68,** 27–35 (2006).

34. McLachlan, A. & Dorvlo, A. Global patterns in sandy beach macrobenthic communities. *J. Coast. Res.* **21,** 674–687 (2005).

35. McArdle, S.B. & McLachlan, A. Sand beach ecology: Swash features relevant to the macrofauna. *J. Coast. Res.* **8,** 398–407 (1992).
